# Systematic review of the effect of individual and combined nutrition and exercise interventions on weight, adiposity and metabolic outcomes after delivery: evidence for developing behavioral guidelines for post-partum weight control

**DOI:** 10.1186/1471-2393-14-319

**Published:** 2014-09-10

**Authors:** Alexander Arkin Berger, Rachel Peragallo-Urrutia, Wanda K Nicholson

**Affiliations:** Public Health Leadership Program, University of North Carolina Gillings Global School of Public Health, 135 Dauer Drive, Chapel Hill, NC USA; Department of Obstetrics and Gynecology, University of North Carolina School of Medicine, 321 South Columbia Street, Chapel Hill, NC USA; Department of Obstetrics & Gynecology, Division of Women’s Primary Health, 3027 Old Clinic Building, CB # 7570, Chapel Hill, NC USA; Diabetes and Obesity Core, Center for Women’s Health Research, University of North Carolina School of Medicine, 3027 Old Clinic Building, CB # 7570, Chapel Hill, NC USA

**Keywords:** Systematic review, Obesity, Post-partum weight retention, Randomized trials

## Abstract

**Background:**

Post-partum weight retention contributes to the risk of chronic obesity and metabolic alterations. We conducted a systematic review of randomized controlled trials (RCTs) on the effect of post-partum nutrition and exercise interventions on weight loss and metabolic outcomes. DATA SOURCES: Four electronic databases were searched from inception to January, 2012. Two investigators reviewed titles and abstracts, performed data abstraction on full articles and assessed study quality.

**Methods:**

We included RCTs comparing nutrition, exercise or combined nutrition and exercise interventions with a control condition. Thirteen studies met our inclusion criteria (N = 1,310 participants). Data were abstracted on study characteristics, intervention components, enrollment period, and length of follow-up. Outcomes of interest included weight, adiposity, cardio-metabolic measures (glucose, lipids) and obesity-related inflammatory markers.

**Results:**

Nine trials compared combined interventions to standard post-partum care; three trials assessed the effect of exercise interventions, one trial evaluated a nutrition-only intervention. Four good quality RCTs on combined interventions had inconsistent findings, with the larger RCT (N = 450) reporting no difference in weight between groups. Four fair-to good quality RCTs reported greater weight loss in the combined intervention group vs. standard care, ranging from 0.17 kg to 4.9 kg. Results from exercise only interventions were inconclusive. Evidence for nutrition only interventions was insufficient. There was insufficient evidence for the effect of post-partum interventions on metabolic risk factors and inflammatory biomarkers.

**Conclusions:**

Combined nutrition and exercise interventions can achieve weight loss, but evidence is limited due to a small number of trials and limitations in study design.

**Electronic supplementary material:**

The online version of this article (doi:10.1186/1471-2393-14-319) contains supplementary material, which is available to authorized users.

## Background

Obesity, fueled largely by excessive caloric intake and sedentary lifestyles, contributes to multiple adverse health conditions in women, including diabetes, cardiovascular disease, and certain cancers (endometrial, colon, and postmenopausal breast). More than 78 million adults age 20 years and older in the United States are obese; 41 million are women [1]. The post-partum period may be a critical period for long-term weight gain and development of obesity for women [2, 3]. Physiological changes of childbirth may contribute to weight retention and weight gain [4, 5]. Compared with weight gain during other life intervals, excess weight retained after childbirth appears to be particularly harmful, as evidence suggests that weight retained in the post-partum tends to be distributed centrally, and therefore, may increase the risk of developing chronic disease [2, 6]. Post-partum interventions that target dietary intake and exercise may help women to achieve a healthy weight after delivery and improve their overall health status.

Interventions that integrate exercise or dietary changes have been shown to achieve weight loss, and reduce cardiovascular [7] and diabetes-related risk factors [8] in middle- and older-aged women, though evidence for their efficacy in post-partum women is limited. By reducing weight after delivery, women may be able to enter a subsequent pregnancy at a healthier weight and reduce their risk of obesity-related pregnancy complications [9–11]. Alternatively, if a woman has completed childbearing, achieving a healthy weight after delivery may help to improve long-term health through the effects on metabolic and obesity-related biomarkers. The American Congress of Obstetricians and Gynecologists [12] provides general recommendations for post-partum physical activity, but there are no current evidence-based U.S. guidelines for diet or exercise interventions to help women achieve a healthy weight after delivery and improve.

A recent systematic review of eight studies [13], suggests that diet and exercise interventions can reduce postpartum weight retention. However, there was no assessment of the effects of lifestyle interventions on metabolic risk factors, such as glucose and lipid levels or adipokines. Prior research suggests that weight loss improves long-term health outcomes through favorable effects on glucose metabolism, lipid aberrations and adipokines. Adipocytokines, such as adiponectin and C-reactive protein, and leptin are inflammatory proteins that have been linked to obesity and type 2 diabetes [14–17]. Adiponectin levels are lower in subjects with obesity and type 2 diabetes; leptin and c-reactive protein correlate positively with body mass index, insulin resistance and diabetes [18, 19]. Elucidating the effects of lifestyle modification on these biomarkers is important to perinatal health providers as it can help them to better inform patients about the potential benefits of postpartum weight loss programs. Additionally, there was little to no data on potential harms of post-partum diet or exercise interventions. The RCTs included in the review were largely limited to combined diet and exercise interventions. Relevant data on the effects of diet and/or exercise on weight and the effects of weight change on obesity-related biomarkers is needed to foster the development of evidence based guidelines for weight loss in the postpartum period.

We conducted a systematic review of the literature to identify randomized trials to assess the benefits and harms of post-partum behavioral weight management interventions that included nutrition, exercise, or combined nutrition and exercise components. The primary questions were 1) Do post-partum behavioral weight management interventions (nutrition, exercise or combined nutrition and exercise) lead to a reduction in weight, body mass index (BMI) or adiposity in women who were overweight or obese at the time of conception or in early pregnancy? 2) Do post-partum behavioral weight management interventions lead to other positive outcomes (e.g. improved glucose and insulin, levels of inflammatory biomarkers)? and 3) what are the adverse effects of post-partum behavioral weight management interventions attempting to reduce weight and improve health outcomes?

## Methods

A preliminary protocol was designed and independently reviewed. The protocol included the literature search strategy, the selection process for choosing studies, the method and forms to be used for extracting data, assessing the quality of studies and the methods for data synthesis and analysis.

### Criteria for considering studies for this review

#### Types of studies

We considered clinical trials that met our pre-determined list of inclusion criteria that were reviewed and approved as part of the preliminary protocol. We included trials that had a least one intervention and one comparison group, which could include a control group, usual care group or alternative behavioral intervention group. Studies without a comparison group were not included.

#### Types of participants

We included studies which enrolled participants with the following factors: women who were postpartum, enrolled in the study in the early postpartum period (up to 12 weeks following delivery); women from any geographical and racial/ethnic background.

### Types of interventions

Interventions that included diet, exercise or diet plus exercise components, took place in the postpartum period and were of at least 4 weeks in duration with the goal of reducing postpartum weight were included in this review. We only considered interventions that included behavioral modifications. Studies that focused on weight loss medications or surgical therapy (e.g. bariatric surgery) for weight loss were not included in the review. Interventions could be conducted in any setting, including primary care settings and hospitals or in community settings.

#### Types of outcome measures

The primary outcome was weight. Additional outcomes of interest for weight and adiposity included percentage of weight loss, proportion of women returning to pre-pregnancy weight, and measures of adiposity, including abdominal (waist) circumference [20], waist-to-hip ratio, and skinfold thickness, and any reported adverse events of the intervention. Cardio-metabolic outcomes included lipids high-density lipoprotein cholesterol [HDL-C], low-density lipoprotein cholesterol [LDL-C], triglycerides), glucose and glycosylated hemoglobin (HbA1c). Obesity-related biomarkers, such as c-reactive protein, interleukin-6, leptin and adiponectin.

### Search methods for identification of studies

A comprehensive search strategy was developed (Search strategy in Additional file 1). We searched 4 electronic databases, MEDLINE, EMBASE, The Cochrane Central Register of Controlled Trials, and the Cumulative Index to Nursing and Allied Health Literature for English-only articles from inception through January 2012. Search terms included MeSH and text terms for post-partum, diet and exercise, behavioral interventions and weight loss. The search strategy included selected index terms, key words and free text terms such as:

“Postpartum period*, postpartum, post partum, post pregnancy, intervention, behavior, *behavioral, life style*, lifestyle, life style, “Exercise Therapy”, exercise*, diet* or exercise therapy* or exercise*

We conducted quality audits to confirm that studies identified through prior literature review and discussions with experts in the field were identified by the search terms. We hand-searched 15 journals that were most likely to publish articles on post-partum weight loss interventions from December 2011 through January 2012 and scanned reference lists from included articles and relevant review articles. Subsequently, we conducted a bridge search from January, 2012 to May, 2013 (See PRISMA checklist in Additional file 2).

### Data collection and analysis

#### Selection of studies

Articles were rejected if they met one or more of the following exclusion criteria: 1) not a RCT; 2) not written in English; 3) did not include human data; 4) no original data (i.e. meeting abstract, editorial, commentary or letter); 5) did not include data on maternal weight loss; 6) less than 50% of the sample was enrolled within one year post-partum; 7) did not compare a diet, exercise or combined diet and exercise intervention to a control group; and 8) intervention was less than 4 weeks in duration. When a title/abstract could not be rejected with certainty, the full text of the article was obtained for further evaluation. After non-relevant studies were excluded, potentially relevant studies were assessed independently by two reviewers (AB, RPU). Differences between reviewers’ assessments were resolved by discussion and consensus.

### Data extraction and management

#### Data synthesis

Two reviewers sequentially abstracted information from each articles using standardized forms. Reviewers abstracted information regarding study characteristics, participants, study setting (for example, city or geographical region), study participants (for example, age, race), intervention type (diet, exercise or combinations of each), mode of intervention delivery (for example, mail correspondence, in-person), timing of initiation of the post-partum intervention, duration of the intervention, length of follow-up after the intervention. Differences were resolved through discussion.

### Assessment of study quality, bias and evidence grading

Two investigators (AB, RPU) independently assessed the quality of trials and used items for selection bias, treatments, outcome measures, statistical methods, and loss to follow-up using methods from the United States Preventive Services Task Force [21, 22] and the University of York Centre for Reviews and Dissemination [23]. The quality of each study was rated as good (low risk for bias), fair, or low (high risk for bias).

The quantity, quality, and consistency of the results, the directness of the measures used for each outcome; the precision of the results and the magnitude of effects were graded based on the GRADE (The Grading of Recommendations Assessment, Development and Evaluation) working group criteria [24]. “High” strength of evidence indicates that the evidence probably reflects the true effect, “moderate” strength indicates that further research may change the results, and “low” strength indicates low confidence that the evidence reflects the true effect and further research is very likely to change the result. “Insufficient” evidence indicates that no studies met our inclusion criteria for the comparison for a given outcome.

### Data synthesis

We conducted a qualitative summary for each outcome by intervention category (diet and exercise, exercise-only and diet-only). We were unable to conduct a meta-analysis of the primary outcome of weight or any secondary outcomes because of heterogeneity in the population studied, variation in study duration and intervention components, and mode of delivery of the intervention components.

## Results

### Results of the search

**Figure 1:**
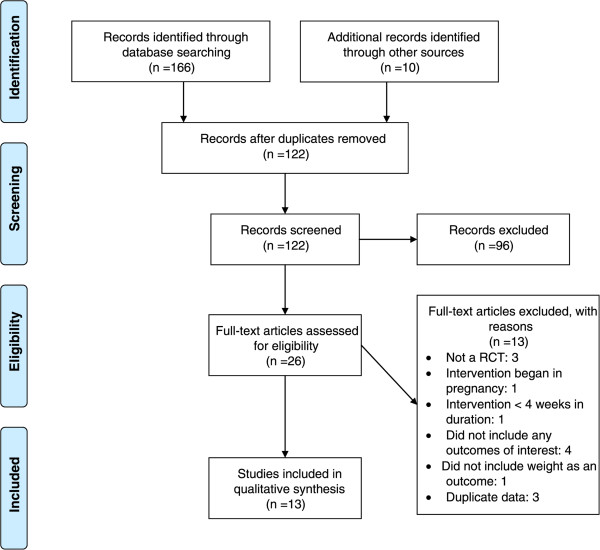
**Summary of the literature search and review process (number of articles).** *Total may exceed the number in the corresponding box because articles could be excluded for more than one reason at this level. CINAHL (Cumulative Index to Allied Health and Nursing Literature).

We retrieved a total of 122 unique citations from electronic databases, hand-searching, and a bridge search (Figure 1). After abstract and full article review, we identified 13 unique publications related to the benefits and harms of behavioral interventions for post-partum weight loss; nine RCTs assessed the effects of combined diet and exercise interventions, (N = 1,015 participants); three RCTs compared exercise-only interventions to usual care (N = 139 participants); one RCT compared a diet-only intervention to usual care (N = 151 participants).

### Description of 13 included trials

Thirteen RCTs were published between 1998 and May 2013 and were conducted in various countries (Table 1). Nine trials were conducted in the United States; one in Taiwan [25], one in Iran [26], one in Greece [27] one in Sweden [20] and one in the United Kingdom [28]. The trials also compared interventions using different modes of delivery. Among the trials of combined interventions, 3 RCTs compared an in-person dietary and exercise intervention to usual care [20, 29]. Bertz and colleagues [30], provided an in-person intervention with dietary modifications aimed at reducing caloric intake by 500 kcal/day and complete 45 minutes of exercise. Huang [25] provided individualized diet and exercise plans. Ostbye and co-authors [29] provided an in-person intervention coupled with telephone calls. Three RCTs compared in-person nutrition and exercise interventions to usual care plus an informational pamphlet [28, 31] or structured handout [32]. One trial [33] compared a diet plus moderate or low intensity in-person intervention with historical controls.

**Table 1:** **Characteristics of 13 RCTs of postpartum nutrition, exercise and combined interventions**

Author, yr, Country	Description of treatment arms	*Enrollment period; **duration	Sample population, N	Age (y)	Race/ethnicity	Study quality
**Nutrition and Exercise Interventions**						
Bertz, 2012, Sweden, in-person [30]	Intervention (I): 3 intervention arms and 1 control arm (D) dietary modifications reduce caloric intake by 500Kcal/day; (E): exercise only- exercise to achieve 45 min brisk walk 4d/week; (DE): combined diet and exercise; Control (C): Usual care	10-14 weeks postpartum; 12 weeks	63 lactating, overweight/obese primiparous; N = 68	33	Swedish	Good
Colleran, 2012,US, in-person [32]	Intervention (I): postpartum counseling on diet and exercise plus on-line resources; Control (C): Usual care + diet and nutrition print handouts	4-6 weeks;16 weeks	31 women, ≥23 years and < 37 years, singleton pregnancy	31	NR	Fair-to-good
			(−5.8 ± 3.5 kg) (7%) compared with the *C* (−1.6 ± 5.4 kg) (3%) (*P* = 0.03).			
Leermakers 1998, US, in-person [31]	Intervention (I): Correspondence lessons, group sessions and telephone follow-up Control (C): Usual care + informational brochure	8 mos; 6 mos	Non-lactating postpartum women; N = 90	31	3% non-white	Fair-to-good
O’Toole, 2003 US, in-person [36]	I: Structured diet + exercise with weekly in-person sessions x 12 wks, bi-weekly sessions x 8 wks, and monthly sessions up to 1-year	6 wks-6 mos; 6–10 mos	Postpartum women who were overweight or obese prior to pregnancy; N = 40	31	1 AA	Good
	C: One in-person session followed by self-directed program					
Craigie 2011, United Kingdom, in-person [28]	I: Information pamphlet + two face to face counseling sessions, with telephone follow-up for reinforcement and resources C: Information pamphlet	6-18 mos; 3 mos	Low income, overweight and obese postpartum women; N = 52	30	3 non-white participants	Fair
Huang, 2011, Taiwan, in-person [25]	I: Individualized dietary and physical activity plans, including 6 pregnancy sessions and 3 postpartum sessions C: Usual care	1 day; 6 mos	Pregnant and postpartum women Taiwanese women; 1 day postpartum; N = 189	32	Taiwanese	Fair-to-good
Lovelady 2000, US, in-person [37]	I: Caloric restriction and exercise intervention, including 4 exercise sessions, lasting 43 minutes with goal of 65-80% heart rate C: Usual care	5 wks; 2.5 mos	Overweight postpartum women with BMI 25–30 kg/m2, exclusively breastfeeding; N = 40	32	3.5% AA	Fair-to-good
Ostbye, 2009, US, in-person [29]	I: 8 healthy eating classes, 10 physical activity classes and 6 telephone counseling sessions over 9 mos. C: Usual care	2 mos; 9 mos	Overweight or obese postpartum women; N = 450	31	I: 45% AA	Good
					C:45% AA	
Davenport, 2011, US, in-person [33]	I: Diet + low intensity exercise	8 wks; 4 mos	Overweight or obese women who retained > 5 kg after delivery; N = 60	33	Intervention groups: 85-90% white	Fair-to-good
	I: Diet + moderate intensity exercise					
					No AA	
	C: Historical controls matched for age, BMI, and parity				Other race: NR	
**Exercise-only Interventions**						
Zourladani, 2011, Greece [15]	I: Instructor led 1-hour exercise class with aerobic activity and strength training 3 times per wk for 12 wks.	4-6 wks; 3 mos	Primiparous postpartum women; N = 40	31	Greek	Fair
	C: No intervention					
Maturi, 2011, Iran [14]	Tailored pedometer-based walking program with baseline counseling session, cell phone and text reminders and telephone feedback; C: Usual Care	6wks-6mos; 3 mos	Lactating, normal or overweight postpartum women; N = 66	25	Iranian	Fair-to-poor
Dewey, 1994, US [34]	I: Individually tailored and supervised aerobic activity to achieve 60-70% heart rate reserve. 45 min-5 times a wk	6-8 wks; 3 mos	Exclusively breastfeeding postpartum women; N = 33	30	No AA	Fair-to-good
	C: No intervention					
**Nutrition-only Interventions**						
Krummel, 2010, US [23]	I: Counseling with dietitian, and 10 facilitated discussion groups, monthly personalized feedback on self-monitoring records	30 wks; 12 mos	Postpartum women enrolled in WIC; N = 151	27	10% non-white;	Fair-to-poor
	C: Self directed					

*Postpartum enrollment period; **duration of postpartum intervention. NR = not reported; BMI = body mass index; AA = African American; mos = months; wks = weeks; WIC = Women’s, Infant, Children’s program.

There were exercise-only RCTs [26, 27, 34]. One trial included in-person structured aerobic and strength training sessions [27]. A second RCT included a pedometer-based walking program with text reminders and telephone feedback [26]. Another trial consisted of individually tailored and in-person supervised aerobic training [34]. A nutrition-only trial [35] compared the effect of individual nutritional counseling and facilitated group sessions with standard post-partum care.

The enrollment period for the 13 trials ranged from one day to six months after delivery. The duration of the interventions was 3 to 9 months. Among the nine trials comparing combined diet and exercise interventions [20, 25, 28, 32, 33, 36–38] with standard care, attrition rates varied widely. Two of these trials [31, 37] reported differential loss to follow up between the intervention and standard care groups. Two trials did not discuss attrition rates [25, 28]. For the exercise-only studies [26, 27, 34], loss to follow-up rates ranged from 5-13%.

### Quality of included trials

There were three good quality RCTs [20, 36, 38] with broad applicability to the general population of post-partum women. There were six fair-to-good quality trials [27, 31, 32, 34, 35, 37]. Only three trials [28, 31, 38] discussed or reported an intention to treat analysis [31, 38].

### Results of included trials

#### Nutrition and exercise interventions

Nine trials compared the effects of post-partum of diet and exercise interventions compared to standard post-partum care on weight (Table 2). Seven of the trials [20, 28, 32, 33, 36, 37] reported greater post-partum weight loss among women in the intervention group compared to those in the usual care group. One trial compared a diet-only, exercise-only and combined diet and exercise intervention to usual care [20]. Ostbye and colleagues [39] reported findings from the largest RCT, in which they randomly assigned 450 post-partum women to receive a combined diet and exercise intervention or standard care. They reported no statistically significant differences in mean weight loss between the two groups. Two trials [33, 37] evaluated body mass index (BMI) and reported a statistically significant difference between the intervention and standard care groups. Leermakers and colleagues [31] found a statistically significantly higher percentage weight loss (10% versus 5.8%; p < 0.04) and proportion of women returning to their pre-pregnancy weight (33% versus 11.5%, p < 0.05) (data not shown) in the intervention group compared to the standard care group. There were no statistically significant differences in abdominal circumference between women in a diet and exercise intervention and those in usual care.

**Table 2:** **Effect of postpartum nutrition and exercise interventions on maternal weight, adiposity**

Author, yr	Weight (kg) SD	BMI (kg/m ^2^)	Adiposity
Nutrition and Exercise interventions			
Bertz (2012) [30]	Weight (12 weeks)		Fat mass:
	D: −8.3 ± 3.0	D: −2.9 ± 1.5	D: −6.9 ± 3.4
	E: −2.4 ± 3.2	E: −0.8 ± 1.0	E: −1.8 ± 3.0
	DE: −6.9 ± 3.0	DE: −2.5 1.0	DE: −6.2 ± 3.1
	C: −0.8 ± 3.0; P < 0.001	C: −0.3 1.1; P < 0.001	C: −0.7 ± 3.1
	Weight (1 year):		1 year:
	D: −10.2 ± 5.7	D: −3.6 ± 2.0	D: −9.2 ± 5.6
	E: −2.7 ± 5.9	E: −0.9 ± 2.0	E: −2.5 ± 5.9
	DE: −7.3 ± 6.3	DE: −2.6 ± 2.2	DE: −6.0 ± 7.0
	C: −0.9 ± 6.6; P < 0.001	C: −0.3 ± 2.4; P < 0.001	C: −1.8 ± 6.2
Colleran (2012) [32]	I: −5.8 (SE:3.5)	I: 0.9 (SE 1.5)	
	C: −1.6 (SE:5.4);	C: 2.1 (SE1.4);	
	P <0.01	P <0.01	
Leermakers (1998) [31]	I: −7.8 ± 4.5		
	C: −4.9 ± 5.4		
	(P^a^ = 0.03)		
Lovelady (2000) [37]	I: −4.8 ± 1.7	I: −1.8 ± 0.6	Skinfold thickness; all 6 measures (P < 0.01)^b^
	C: −0.8 ± 2.3	C: −0.3 ± 0.9 (P < 0.01)	
	(P < 0.001)		
O’Toole (2003) [36]	I: −7.3		
	C: −1.3		
	(P < 0.05)		
	SD-NR		
Ostbye (2009) [29]	I: −0.9 ± 5.1		
	C: −0.36 ± 4.9		
	(P = 0.25)		
Craigie (2011) [28]	I: −1.6 ± 2.0		Waist circumference
	C:0.2 ± 2.2		I: −4.4 ± 3.5
	(P = 0.018)		C: −2.8 ± 4.5
			(P = 0.23)
			% body fat
			I: −1.5 ± 0.8
			C: −0.5 ± 1.4
			(P = 0.32)
Huang (2011) [25]	I: −11.21		
	C: −11.04		
	(P-value NR)		
	SD-NR		
Davenport (2011) [33]	I: −5.0 ± 2.9	I: −1.8	Waist-to-Hip Ratio
	Diet + moderate intensity exercise;	moderate intensity	I: −0.05
		I: −1.5	I: −0.03
	I: −4.2 ± 4.0	low intensity	C: 0.0
	Diet + low intensity exercise;	C: 0.0	SD:NR
	Historical controls:	SD-NR	(P < 0.05)^a^
	−0.1 ± 3.3	(P < 0.05)^a^	
	(P < 0.01)^a^		
**Exercise-only interventions**			
Dewey (1994) [34]	I: −1.6		
	C: −1.6		
	(p > 0.05)		
	SD-NR		
Zourladani (2011) [27]	I: −3.3		
	C: −1.3		
	(P = 0.667)		
	SD-NR		
Maturi (2011) [26]	I: −2.1	I: −0.81	Waist-to-Hip Ratio
	C: 0	C: 0.1	I: −0.02
	(P < 0.001)	(P < 0.001)	C: 0.01
	SD-NR	SD-NR	(P = 0.02)
			SD-NR
**Diet-only interventions**			
Krummel (2010) [35]	I: −2.9 ± 11.8	I: −0.54 ± 1.9	
	C: −2.9 ± 10.7	C: −0.54 ± 1.8 (P > 0.05)	
	(P > 0.05)		

SD = Standard Deviation; NR = Not reported ^a^P-values reflect relationship between control and the medium and low intensity exercise groups. ^b^Skinfold thickness measures include triceps, subscapular area, mid-axillary line, abdomen, thigh and suprailiac area.

In a 3-arm RCT, Davenport and colleagues [33] reported outcomes among participants completing a nutrition + low intensity intervention and a nutrition + moderate intensity to historical controls matched for age, BMI and parity. There was statistically significant greater weight loss (−5.0 ± 2.9 moderate intensity, -4.2 ± 4.0 low intensity, respectively, compared to controls −0.1 ± 3.3; P < 0.01) and lower waist-to-hip ratios (−0.05 moderate intensity, −0.03 low intensity vs. 0.00 control; P < 0.05) among participants in the intervention groups compared to historical controls matched for age, BMI and parity (Table 2). Davenport and co-investigators [33] also reported on differences in cardio-metabolic and biomarker outcomes between the three groups. There were small, but statistically significantly lower fasting glucose (I: −0.07 mg/dl, I: −0.5 mg/dl, and C: 0.7 mg/dl; P, 0.05) and adiponectin (I: 3,610 ng/ml, :3,890 ng/ml, C: 1575 ng/ml; P < 0.05) levels in the nutrition plus moderate and nutrition plus low intensity exercise intervention groups, respectively, compared to historical controls. There were no available data on C-reactive protein or leptin levels.

#### Exercise-only interventions

Maturi and colleagues [26] reported greater weight loss in the intervention group compared to the usual care group. Also, women in the intervention group had a lower BMI (p < 0.001) and waist-to-hip ratio (p = 0.02) compared to those in the usual care group. Two smaller RCTs [27, 34] reported no statistically significant differences in average weight loss. Maturi and colleagues [26] reported waist-to-hip ratios that were lower among women receiving an individualized pedometer-based program compared to those in usual care.

#### Nutrition-only interventions

One trial [23] met our inclusion criteria. Krummel and colleagues [35] reported no difference in weight or BMI between women in a diet-only intervention and those receiving usual care.

### Adverse effects of post-partum behavioral interventions

We found little data on harms of post-partum behavioral interventions. There were no reports of adverse maternal events or deaths related to the interventions. Dewey and colleagues reported no change in milk volume and composition among women enrolled in an exercise-only intervention compared to usual care [34].

### Overall strength of evidence

Taking into account the number of RCTs, study quality inconsistency in outcome measures across studies and inconsistent study findings, we graded the overall strength of evidence as low for the effect of post-partum lifestyle interventions on the reported maternal outcomes (Table 3). The duration of interventions was generally short (≤9 months). Little to no information was provided on treatment harms or adverse events.

**Table 3:** **Strength of evidence for effect of postpartum lifestyle interventions on weight, adiposity, metabolic and biological markers**

Outcome	Strength of evidence	Intervention	Conclusions
Weight	Low	Nutrition and Exercise	Four good quality RCTs had inconsistent findings. Three of the four fair-to-good quality studies reported greater weight loss in the intervention group compared to standard postpartum care, but the RCTs were short (≤9 months) in duration and had limited generalizability to racial and ethnic minority groups.
	Low	Exercise only	Results were inconclusive for comparison of exercise-only interventions with standard postpartum care, with low risk for bias, but moderate imprecision and inconsistency in the dire study findings.
	Insufficient	Nutrition only	Only one RCT for comparison of diet-only with standard postpartum care, with high attrition rates and differential loss to follow-up between treatment groups.
Adiposity	Low	All interventions	Few studies included adiposity and there is inconsistency in adiposity measures across studies. One comparison showed a reduction in skinfold thickness; two comparisons reported a statistically significant reduction in waist-to-hip ratios, but estimate of effect were imprecise due to small sample sizes.
Cardio-metabolic	Insufficient	All interventions	One RCT compared lipid and glucose levels between women receiving a nutrition and exercise intervention and historical controls
Biological markers	Insufficient	All interventions	Only one RCT was included for the outcome of adiponectin. Findings from the intervention group were compared to non-randomized historical controls.

## Discussion

We found an overall low strength of evidence for post-partum interventions on weight loss. Three good quality trials [36, 38] comparing combined nutrition and exercise interventions with standard post-partum care reported inconsistent findings; four of five fair-to-good quality RCTs reported small to modest weight loss (0.6 kg to 4 kg) among post-partum women with the use of combined nutrition and exercise interventions. Evidence from three exercise-only RCTs is inconclusive. Evidence for exclusively dietary interventions on weight loss is insufficient. Additionally, evidence for the effect of post-partum lifestyle interventions on maternal adiposity, cardio-metabolic outcomes and biological markers is insufficient. Based on the number and quality of studies reviewed, we conclude that combined nutrition and exercise intervention can achieve post-partum weight loss, but we are unable to draw substantial conclusions of effectiveness due to the inconsistency in findings from the three higher quality RCTs [20, 36, 38] and particularly the lack of differences in weight reported by the largest trial to date by Ostbye and colleagues [38].

This systematic review adds to the literature on post-partum weight management interventions in several relevant areas. First, we include a broader scope of studies that included four additional RCTs (two additional combined interventions, one exercise-only and one nutrition-only). Second, we expanded the clinical outcomes of the review to include important cardio-metabolic health outcomes and obesity-related biomarkers shown to be responsive to modifications in nutritional intake and exercise in earlier studies. Post-partum interventions that promote a favorable biomarker profile may reduce the risk of chronic obesity and the downstream consequences of cardiovascular disease, diabetes and obesity-related cancers (e.g. breast, endometrial, colon). Also we included a review of potential harms related to post-partum behavioral interventions. Some investigations have suggested that maternal weight loss can diminish the quality of lactation, but based on limited available data in the clinical trials reviewed [34], we found no evidence for an increase in adverse maternal events. We found no evidence for an increase in adverse maternal or infant outcomes.

There are few evidence-based guidelines for post-partum nutrition and exercise (Table 4). In 2009, the American Congress of Obstetricians and Gynecologists reaffirmed earlier recommendations for exercise in the post-partum period and advocated that pre-pregnancy exercise may be gradually resumed as soon as medically safe [39]. Guidelines from the Canadian Society for Exercise Physiologists [40] suggest that most women can return to their normal exercise programs after receiving medical clearance at their first postnatal check-up [30]. In the United Kingdom, guidelines governing dietary interventions and physical activity interventions for weight management after pregnancy were developed by the National Institute for Health and Clinical Excellence (NICE) [41]. These guidelines were developed based on a review of the efficacy cost-effectiveness of interventions, fieldwork data and comments from stakeholders and experts and include targeted goals, such as a low-fat diet, regular physical activity, and identifying and addressing barriers to change.

**Table 4:** **Comparison of National Institute of Clinical Excellence and American Congress of Obstetricians and Gynecologists**

Recommendations		
	All Post-partum Women	Post-partum women with BMI ≥ 30
NICE	6–8-week postnatal check	6-8 week postnatal check
	In women who are overweight, obese or who have concerns about their weight, ask if they would like any further advice and support now – or later. If yes, make an appointment within the next 6 months for advice and support.	Explain the increased risks that being obese poses to them and, if they become pregnant again, their unborn child.
	Provide current advice about how to lose weight after childbirth.	Encourage them to lose weight.
	Ensure women have a realistic expectation of the time it will take to lose weight gained during pregnancy.	Offer a structured weight-loss program or a referral to a dietitian or an appropriately trained health professional.
	Discuss benefits of a healthy diet and regular physical activity,	
	Advice on healthy eating and physical activity should be tailored to individual patient circumstances.	
		Provide Women who are not yet ready to lose weight with information about where they can get support when they are ready.
	Advise women to seek information from reliable sources	
	Provide details of appropriate community-based services	
	Encourage women to breastfeed.	
		Use evidence-based behavior change techniques to motivate and support women to lose weight.
	Provide advice on recreational exercise from the Royal College of Obstetrics and Gynecology:	
	A mild exercise program of walking and stretching may begin immediately; after complicated deliveries, or lower segment caesareans, a medical provider should be consulted before resuming pre-pregnancy levels of physical activity.	Encourage breastfeeding
	Emphasize importance of physical activities that can be built into daily life.	
ACOG	Rapid return to pre-pregnancy activities is acceptable after an uncomplicated pregnancy and delivery	No recommendations based on BMI
	Moderate weight reduction after delivery does not interfere with lactation or neonatal weight	
	Postpartum exercise may help to reduce postpartum depression symptoms	
	Refer to weight specialist before the next pregnancy	
	Discuss healthy lifestyle behaviors at each visit.	

Adapted from the National Institute of Health and Clinical Excellence [27] and the American Congress of Obstetricians and Gynecologists [26].

There are limitations that deserve attention. Limitations to the current review include possible publication bias. Large-scale studies that showed no differences between groups can remain unpublished and affect the ability to draw meaningful conclusions. Also, we only included articles written in English which may have contributed to reporting bias. Additionally, few measures of maternal adiposity or cardio-metabolic outcomes were included in two or more studies. There were several fair quality trials with limited information on steps to ensure adherence to the intervention protocol. This limitation in intervention fidelity further reduces the overall strength of available evidence. There were little to no data reported on which components of the intervention were most often used by participants and which components were most associated with the desired outcome. Additionally, the extent or type of care provided to the comparator group varied between studies making it difficult to draw meaningful conclusions from some studies. Trials compared the intervention group to standard post-partum care, self-directed groups and control conditions in which no detailed descriptions were provided. Only three trials [35, 36, 38] included an intervention that was longer than 6 months in duration, limiting the ability to assess the effects of interventions on long-term weight loss and other health outcomes. There was little data on potential harms of interventions, such as adverse clinical outcomes or participant time and effort.

## Conclusion

This review has several relevant implications for clinical practice and future research. Although limited, obstetricians and primary care providers now have evidence to support referral to counseling and behavioral interventions for overweight and obese women after delivery. Providers will need to communicate these findings to patients, create partnerships with programs and facilities offering behavioral interventions, and facilitate timely referral of their patients to these programs. In this fashion, post-partum women can receive these interventions in collaborative, multidisciplinary settings. Future studies that include a larger sample size and are adequately powered to detect meaningful differences in weight between treatment and control groups, consistent outcome measures of maternal adiposity as well as levels of glucose and obesity-related biomarkers are needed to broaden our knowledge of effectiveness and potential harms of post-partum nutrition and exercise interventions. Studies with well-developed protocols to ensure intervention fidelity can provide important information on the effectiveness of individual intervention components. Future studies should include interventions lasting 9 months or more to provide data on longer term outcomes. Finally, studies should collect and report data on any adverse events of post-partum weight management. On-going RCTs registered with ClinicalTrials.gov [42] may provide additional insight on the effect of behavioral interventions on weight and relevant metabolic biomarkers.

## Electronic supplementary material

Additional file 1: **Search strategy.** (DOCX 14 KB)

Additional file 2: **PRISMA checklist.** (DOC 64 KB)

## Abbreviations

BMI: Body mass index
GRADE: The grading of recommendations assessment, development and evaluation
HbA1C: Hemoglobin A1C
HDL-C: High-density lipoprotein cholesterol
LDL-C: Low-density lipoprotein cholesterol
NICE: National institute for health and clinical excellence
RCT: Randomized controlled trials.
