# Supplementary material for: Systematic review of the effect of individual and combined nutrition and exercise interventions on weight, adiposity and metabolic outcomes after delivery: evidence for developing behavioral guidelines for post-partum weight control
Source: BMC Pregnancy Childbirth. 2014 Sep 10;14:319. doi: 10.1186/1471-2393-14-319 (PMC4176850; doi:10.1186/1471-2393-14-319)
Supplement: Supplementary file 1 — Additional file 1: Search strategy. (DOCX 14 KB) [file 12884_2013_1195_MOESM1_ESM.docx]

**Additional file 1. MEDLINE literature search terms**

| **Population** | "Post Partum Period"[Mesh] OR "Post-Pregnancy" |
| --- | --- |
| **Interventions** | "Postpartum period [MeSH]" OR "postpartum" OR “post partum” OR "Post pregnancy"  AND  “Intervention” OR “behavior” [MeSH] OR “behavioral” OR "Life Style"[Mesh] OR "lifestyle" OR "life style" or "Exercise Therapy"[MeSH] OR "Exercise"[MeSH] OR "Diet"[Mesh] or “Exercise Therapy” [MeSH] or “Exercise” [MeSH]  AND  “Body Mass Index” [MeSH] OR “Weight Loss” [MeSH] OR “Hip AND Waist AND Ratio” OR “Abdominal and Circumference” OR “Skinfold Thickness” [MeSH] |
| **Limits** | Humans  Female  Randomized Controlled Trial  English language |
